# Epidemiology of Hepatitis C Virus in HIV Patients from West Mexico: Implications for Controlling and Preventing Viral Hepatitis

**DOI:** 10.3390/pathogens13050360

**Published:** 2024-04-27

**Authors:** Alexis Jose-Abrego, Maria E. Trujillo-Trujillo, Saul Laguna-Meraz, Sonia Roman, Arturo Panduro

**Affiliations:** 1 Department of Genomic Medicine in Hepatology, Civil Hospital of Guadalajara, “Fray Antonio Alcalde”, Guadalajara 44280, Jalisco, Mexico; alexisjoseabiology@gmail.com (A.J.-A.); kaia_karissa@hotmail.com (M.E.T.-T.); s.laguna.meraz@gmail.com (S.L.-M.); sonia.roman@academicos.udg.mx (S.R.); 2 Health Sciences Center, University of Guadalajara, Guadalajara 44340, Jalisco, Mexico

**Keywords:** hepatitis C virus, HIV, prevalence, risk factors, genotypes/subtypes, liver damage, mutations

## Abstract

The complex epidemiology of hepatitis C virus (HCV) infection among human immunodeficiency virus (HIV) patients in West Mexico remains poorly understood. Thus, this study aimed to investigate the HCV prevalence, HCV-associated risk factors, and HCV genotypes/subtypes and assess their impacts on liver fibrosis in 294 HIV patients (median age: 38 years; 88.1% male). HCV RNA was extracted and amplified by PCR. Hepatic fibrosis was assessed using three noninvasive methods: transient elastography (TE), the aspartate aminotransferase (AST)-to-platelets ratio index score (APRI), and the fibrosis-4 score (FIB4). Patients with liver stiffness of ≥9.3 Kpa were considered to have advanced liver fibrosis. HCV genotypes/subtypes were determined by line probe assay (LiPA) or Sanger sequencing. The prevalence of HIV/HCV infection was 36.4% and was associated with injection drug use (odds ratio (OR) = 13.2; 95% confidence interval (CI) = 5.9–33.6; *p* < 0.001), imprisonment (OR = 3.0; 95% CI = 1.7–5.4; *p* < 0.001), the onset of sexual life (OR = 2.6; 95% CI = 1.5–4.5; *p* < 0.001), blood transfusion (OR = 2.5; 95% CI = 1.5–4.2; *p* = 0.001), tattooing (OR = 2.4; 95% CI = 1.4–3.9; *p* = 0.001), being a sex worker (OR = 2.3; 95% CI = 1.0–5.4; *p* = 0.046), and surgery (OR = 1.7; 95% CI = 1.0–2.7; *p* = 0.042). The HCV subtype distribution was 68.2% for 1a, 15.2% for 3a, 10.6% for 1b, 3.0% for 2b, 1.5% for 2a, and 1.5% for 4a. The advanced liver fibrosis prevalence was highest in patients with HIV/HCV co-infection (47.7%), especially in those with HCV subtype 1a. CD4+ counts, albumin, direct bilirubin, and indirect bilirubin were associated with liver fibrosis. In conclusion, HCV infection had a significant impact on the liver health of Mexican HIV patients, highlighting the need for targeted preventive strategies in this population.

## 1. Introduction

Hepatitis C virus (HCV) is a global infection affecting 58 million people, and approximately 290,000 individuals died in 2019 due to hepatitis C-associated cirrhosis and hepatocellular carcinoma [1]. Specifically, the most severe forms of hepatitis C have been seen in individuals with immunosuppression, such as patients with human immunodeficiency virus (HIV) [2]. Globally, 2.3 million people with HIV are co-infected with HCV (HIV/HCV co-infection) [3] due to the convergence in the transmission routes of both viruses [1]. The highest rate of HIV/HCV co-infection has been reported in Iran at 78.4% [4], while the lowest has been found in Spain at 2.2% [5]. This divergence may indicate that the burden of HIV/HCV co-infection may vary depending on the geographic region, likely due to the prevailing risk factors in each country.

The western region of Mexico comprises the states of Jalisco, Nayarit, Colima, Guerrero, Michoacan, and Sinaloa (West Mexico) [6]. West Mexico serves as a key route for drug trafficking, with heroin, cocaine, and methamphetamines moving from South America to the United States (US) [7]. Furthermore, the presence of powerful cartels influences the availability of oral and injection drugs in the region [7]. This geographic background is important because it is well-known that injection drug use (IDU) is a critical risk factor for contracting HIV and HCV infections [3]. Additionally, due to industrial development, the average income level of people living in West Mexico is higher than in other regions of the country [8]. However, there is also a significant economic disparity, where urban areas accumulate wealth, while rural areas live in poverty [8]. This aspect could impact the prevalence of hepatitis C in this region, given that an association between a low socioeconomic level and an increased risk of HCV infection has been identified [9]. In 2021, the state of Jalisco ranked fourth, with the largest population identifying as part of the lesbian, gay, bisexual, transgender, intersex, and other diverse sexual orientations and gender identities (LGBTI+) community in Mexico, with an estimated 298,270 individuals belonging to this community [10]. It is acknowledged that the HIV/HCV population manifests a high diversity of sexual preferences [11,12]. These characteristics could create favorable conditions to elevate the risk of both HIV and HCV transmission in West Mexico. However, the complex epidemiology of HIV/HCV co-infection is scarcely known in this region. Thus, this study aimed to calculate the prevalence of HIV/HCV co-infection, identify its associated risk factors, determine HCV genotypes/subtypes, and assess the impact of HCV co-infection on liver fibrosis among patients with HIV in West Mexico.

## 2. Materials and Methods

### 2.1. Study Population

Between January 2015 and December 2018, we conducted a cross-sectional study at the Department of Genomic Medicine in Hepatology, the Civil Hospital of Guadalajara, which mainly serves the low-income population in Western Mexico. Eligible participants were adults of ≥18 years of age who had received a confirmed diagnosis of HIV infection at the hospital’s HIV clinic, had a medical history to verify this diagnosis, and were seeking viral hepatitis B/C molecular testing or liver damage diagnosis. All participants were residents of one of the western states of Mexico and demonstrated an understanding of the purpose and risks of this study. Written informed consent was obtained from each participant to ensure clarity and compliance with the study procedures. Sociodemographic data and risk factors for viral hepatitis were collected using a structured questionnaire. 

To calculate the sample size, we used the formula for a proportion, *n* = (*Z*^2^)(*p*)(*q*)/(*d*^2^) [13]—where *n* = the sample size, *Z* = the *Z*-value corresponding to the desired confidence level (1.96 for a 95% confidence level), *p* = the estimated proportion of HIV/HCV co-infection in the hospital population, which was 12.1% in this case [11], *q* = 1 − *p* (the proportion of the population without the characteristic), and d = the margin of error, which was 0.05—substituting these parameters into the formula: *n* = (1.96^2^) (0.121) (1 − 0.121)/(0.05^2^) = 134.9. A minimum sample size of 135 participants was recommended to study HIV/HCV co-infection.

### 2.2. Laboratory Tests and Hepatitis C Serology

Blood samples of 11 mL were drawn using standard venipuncture procedures, and they were subsequently analyzed at the hospital’s general laboratory to assess the levels of liver enzymes, platelets, and CD4+ and CD8+ cells. The CD4+ and CD8+ cell counts were determined using flow cytometry with the Navios instrument (Beckman Coulter Inc., Brea, CA, USA). HCV infection was detected using the anti-HCV enzyme-linked immunosorbent assay (ELISA) method (AxSYM^®^, Abbott Laboratories, Abbott Park, IL, USA), and HCV viral load levels were quantified through real-time PCR (COBAS^®^ AmpliPrep/COBAS^®^ TaqMan^®^ HCV Test, v2.0).

### 2.3. RNA Isolation and Reverse Transcription

In this study, patients who tested positive for HCV using the ELISA method were categorized as HIV/HCV-co-infected. Subsequently, the levels of HCV viral load were assessed, and the RNA-HCV of individuals with a detectable viral load was extracted using the QIAamp Viral RNA Kit method (Qiagen Science, Hilden, Germany), following the manufacturer’s instructions. The extracted RNA was then converted into complementary DNA (cDNA) through a two-step process involving reverse transcriptase and random primers. Initially, we combined 1 µL of random primers (10 µM), 1 µL of dNTPs mix (10 mM), 5 µL of RNA, and 13 µL of PCR-grade water. This mixture was incubated at 65 °C for 5 min. Next, we introduced 4 µL of a buffer (5×), 1 µL of dithiothreitol (0.1 M), 1 µL of RNase Out (40 U/µL), and 1 µL of SuperScript III reverse transcriptase (200 units/µL) (Thermo Fisher Scientific, Carlsbad, CA, USA). The resulting mixture was incubated at 50 °C for 30 min. Subsequently, the reaction was inactivated by heating at 70 °C for 15 min and then stored at −20 °C until use.

### 2.4. HCV Genotyping/Subtyping

#### 2.4.1. Line Probe Assay (LiPA)

HCV genotypes were determined by hybridization line probe assay (LiPA) using the VERSANT HCV Genotype 2.0 kit (Siemens Healthcare Diagnostics, Tarrytown, NY, USA), which simultaneously amplifies the 5’ untranslated region (5’ UTR) and core region through RT-PCR. The RT-PCR products were biotinylated and then hybridized with probes immobilized on nitrocellulose membranes during this procedure. The HCV genotype was determined based on the resulting banding pattern following the manufacturer’s instructions.

#### 2.4.2. DNA Sanger Sequencing

Samples that LIPA could not genotype were analyzed by DNA Sanger sequencing. Initially, the NS5B region was amplified using the forward primer (5’TGATACCCGCTGCTGYTTTGACTC’3, 8258–8639) and reverse primer (5’GTACCTGGTCATAGCCTCCGTC’3, 8618–8639) [14]. The reaction mixture contained 12.5 µL of HotStarTaq Master Mix 2× (comprising 2.5 units of HotStarTaq DNA polymerase, 1× PCR buffer, and 1.5 mM MgCl_2_), 1 µL of the forward primer (10 µM), 1 µL of the reverse primer (10 µM), and 5 µL of cDNA (from the reverse transcription step). The amplification conditions included an initial activation phase at 95 °C for 15 min, followed by a 45-cycle phase (94 °C for 1 min, 60 °C for 1 min, and 72 °C for 2 min), and a final extension phase at 72 °C for 10 min. Then, the products were visualized on 2% agarose gel stained with SybrGreen (Invitrogen, Carlsbad, CA, USA). The labeling reaction was conducted using 1 µL of the purified NS5B-PCR product, 1 µL of the sequencing buffer (5×), 2 µL of the forward or reverse primer (0.8 pmol/µL), 4 µL of nuclease-free water, and 2 µL of BigDye Terminator V3.1 (Applied Biosystems, Foster City, CA, USA). The primers in this reaction were identical to those used in the NS5B-PCR step but at a lower concentration. The cycle sequencing consisted of an initial cycle at 96 °C for 1 min, followed by 24 cycles at 96 °C for 10 s, 50 °C for 5 s, and 60 °C for 4 min. Finally, the process concluded with a step at 4 °C to maintain the sample’s integrity. The sequencing reactions were purified using the ExoSAP-IT PCR clean-up kit (GE Healthcare, Buckinghamshire, UK), and subsequent fragments were analyzed through capillary electrophoresis using the ABI 3130 DNA Genetic Analyzer instrument (Applied Biosystems, Foster City, CA, USA).

Next, the sample sequences were aligned with 52 reference sequences (HCV genotypes 1–6) using the ClustalW method implemented in MEGA software Version 11.0 [15]. To visualize the HCV genotyping/subtyping clusters, a phylogenetic tree was constructed using the maximum likelihood method with the general time-reversible model incorporating gamma distribution and invariable sites (GTR + G + I) and a bootstrap value of 500. All sequenced samples were deposited in GenBank with accession numbers OR610546–OR610580.

### 2.5. Assessment of Liver Fibrosis

Hepatic fibrosis was assessed using three noninvasive methods: transient elastography (TE), the aspartate aminotransferase (AST)-to-platelets ratio index score (APRI), and the fibrosis-4 score (FIB4). The FibroScan instrument (Echosens, Paris, France) was used for transient elastography, and the liver stiffness measurement (LSM) was expressed in kilopascals (kPa). The LSM results were categorized into four stages: F1 (<7.1 kPa), F2 (≥7.1 kPa), F3 (≥9.3 kPa), and F4, indicating cirrhosis (≥12.3 kPa). Moreover, mild liver fibrosis was defined as F1–F2, while advanced liver fibrosis encompassed F3–F4 [16]. The AST and alanine Aminotransferase (ALT) levels were determined using the AU5800 Clinical Chemistry System (Beckman Coulter Inc., Brea, CA, USA), employing its enzymatic method. The APRI score was calculated considering the upper limit of normal (ULN) serum AST level of 40 IU/L in the following equation: [(AST level IU/L)/(AST ULN) × 100]/platelet count 10^9^/L [17,18]. The FIB4 was calculated using the following equation: (age − years) × (AST level IU/L)/(platelet count 10^9^/L) × (ALT)^1/2^ IU/L) [19]. Significant hepatic fibrosis was considered for an APRI value of ≥0.7, while advanced fibrosis was determined for a FIB4 value of ≥3.25 [20,21].

### 2.6. Statistical Analysis

Continuous variables were represented using the median and interquartile range (IQ), while categorical variables were presented as frequencies or percentages. When applicable, comparisons of categorical variables were conducted using the χ^2^ test or Fisher’s exact test. The Shapiro–Wilk test was employed to assess data normality. Non-parametric continuous variables were compared using the Mann–Whitney test. Quantitative value thresholds were determined through receiver operating characteristic (ROC) curve analysis. All statistical analyses were performed using the Statistical Program for Social Sciences software (SPSS 22.0, IBM, Inc., Armonk, NY, USA). A significance level of *p* < 0.05 was considered statistically significant for all tests.

### 2.7. Ethics

This research adhered to the principles outlined in the Helsinki Declaration. This study received approval from the Ethics Committee of the Health Sciences Center at the University of Guadalajara (reference number: CI−07218).

## 3. Results

### 3.1. General Characteristics of the Study Population

Table 1 depicts the sociodemographic and clinical characteristics of the study groups. The study population comprised 294 patients with HIV who exhibited a median HIV viral load of 40 copies/mL. Most were male (88.1%, 259/294), with a median age of 38.0 years, and 35.6% (101/284) of the participants had a high school or university education. The median age of onset of sexual activity was 17.0 years (interquartile range (IQR): 15.0–18.0 years), and 56.9% (160/281) identified as bisexual or homosexual. In this HIV cohort, the prevalence of current or past hepatitis B infection (49.3%; 145/294) was previously described [22,23].

Overall, the prevalence of HCV infection was 36.4% (107/294) among the HIV cohort. Most of the HIV/HCV co-infected patients were identified as heterosexual (54.8%; 57/104), and their educational attainment was significantly lower compared with HIV patients without hepatitis C (education, basic/none: 77.1%, 81/105 vs. 57.0%, 102/179; *p* < 0.001). Also, the levels of alanine aminotransferase (ALT), aspartate aminotransferase (AST), and gamma-glutamyl transferase (GGT) were significantly higher in HIV/HCV co-infected patients than HIV patients without hepatitis C infection (*p* < 0.001). According to the anti-HBc result alone, 31.8% (34/107) of HIV/HCV co-infected patients had prior HBV infection, unbeknownst to them.

### 3.2. HCV by Age Group and Risk Factors

**Figure 1:**
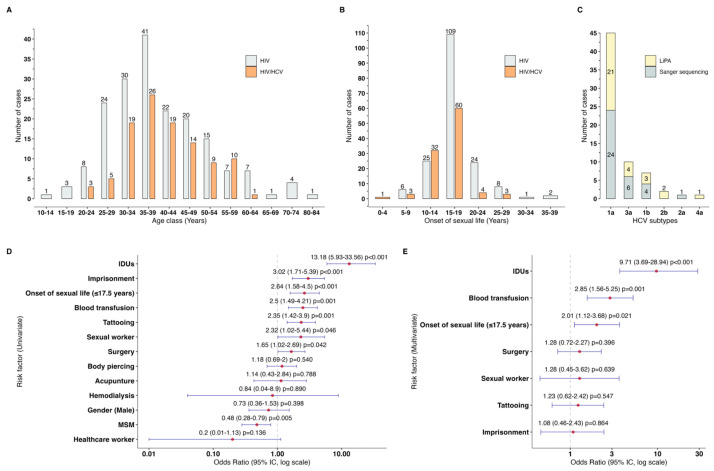
Epidemiology of HCV infection in patients with HIV. Prevalence of HIV and HIV/HCV co-infection by (**A**) age and (**B**) onset of sexual life. (**C**) Distribution of HCV subtypes in HIV population. The forest plots show the main risk factors associated with HIV/HCV co-infection based on (**D**) univariate and (**E**) multivariate analysis. HCV: hepatitis C virus, HIV: human immunodeficiency virus, OR: odds ratio, CI: confidence interval, and LiPA: line probe assay.

As shown in Figure 1A, HIV patients began their co-infection with HCV at the age of 20–24 years. Then, the number of HCV cases increased with age until reaching its maximum peak at 35–39 years of age. After this peak, there was a gradual decline in frequency, and the last cases of HIV/HCV co-infection were seen in the age group of 60–64 years (Figure 1B). The onset of sexual activity for HIV/HCV co-infected patients occurred significantly earlier than for HIV patients without HCV (15.0 years (IQR: 13.0–18.0 years) vs. 18.0 years (IQR: 15.0–19.0) years; *p* < 0.001). Univariate risk factors associated with HIV/HCV co-infection included IDU (OR = 13.2; 95% CI = 5.9–33.6; *p* < 0.001), imprisonment (OR = 3.0; 95% CI = 1.7–5.4; *p* < 0.001), the onset of sexual life at ≤17.5 years (OR = 2.6; 95% CI = 1.5–4.5; *p* < 0.001), blood transfusion (OR = 2.5; 95% CI = 1.5–4.2; *p* = 0.001), tattooing (OR = 2.4; 95% CI = 1.4–3.9; *p* = 0.001), being a sex worker (OR = 2.3; 95% CI = 1.0–5.4; *p* = 0.046), and surgery (OR = 1.7; 95% CI = 1.0–2.7; *p* = 0.042) (Figure 1D). Multivariate analysis revealed that the simultaneous presence of IDU (OR = 9.7; 95% CI = 3.7–28.9; *p* < 0.001), blood transfusion (OR = 2.8; 95% CI = 1.6–6.3; *p* = 0.001), and the onset of sexual activity at ≤17.5 years (OR = 2.0; 95% CI = 1.1–3.9; *p* = 0.021) increased the risk of HIV/HCV co-infection (Figure 1E).

### 3.3. HCV Subtypes in HIV Patients

**Figure 2:**
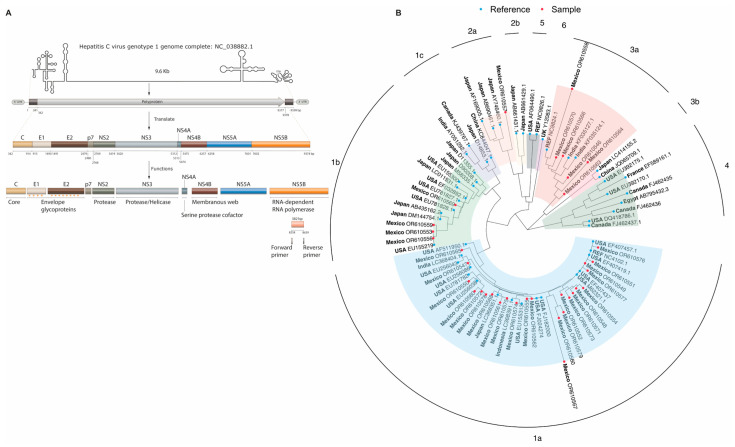
HCV genomic map and the phylogenetic tree utilized for HCV subtyping. In panel (**A**), the complete length of the HCV genome is displayed alongside its primary transcript. This transcript encodes a polyprotein, which undergoes fragmentation to produce the main viral proteins. The pink fragment indicates the specific region utilized for HCV subtyping in this study. Panel (**B**) presents the phylogenetic tree, with sequences labeled by country and GenBank accession number. Blue dots indicate reference samples, while study samples are highlighted in red.

HCV was subtyped in 66 samples from HIV patients (Figure 1C), of which 31 were detected using LiPA, and 35 were identified through phylogenetic analysis (Figure 2). Overall, HCV subtype 1a (68.2%; 45/66) was the most prevalent, followed by 3a (15.2%; 10/66), 1b (10.6%; 7/66), 2b (3.0%; 2/66), 2a (1.5%; 1/66), and 4a (1.5%; 1/66). The distribution of HCV subtypes was similar between the two subtyping methodologies (Figure 1C).

### 3.4. HIV Groups and Liver Damage

**Figure 3:**
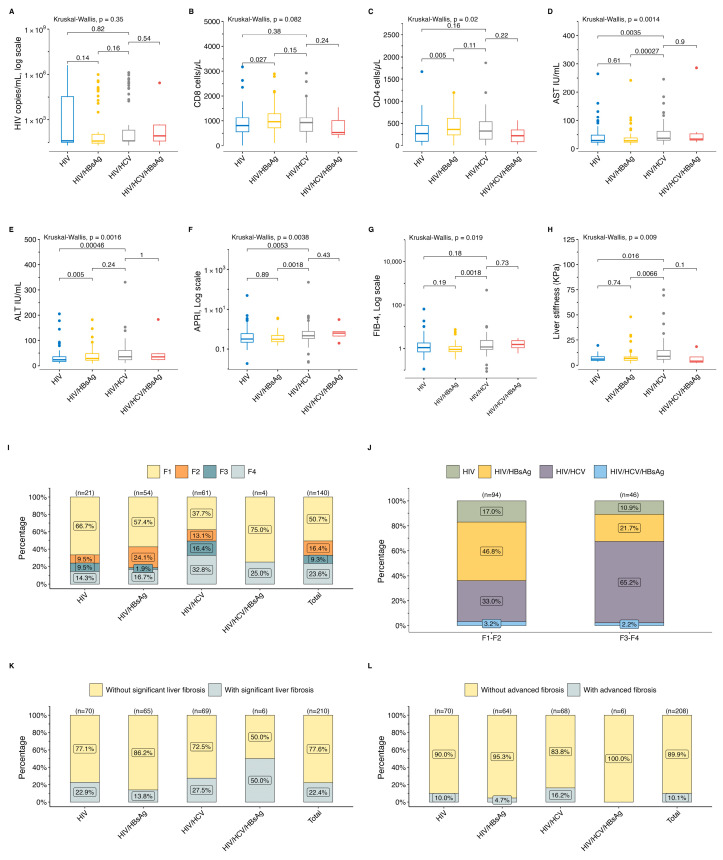
Clinical parameters by study group. (**A**) HIV viral load, (**B**) CD8+ count, and (**C**) CD4+ count. Levels of (**D**) AST, (**E**) ALT, (**F**) APRI, and (**G**) FIB4. (**H**) Liver stiffness measurements. (**I**,**J**) Prevalence of liver fibrosis by study group. Prevalence of liver fibrosis by (**K**) APRI score and (**L**) FIB4. AST: aspartate aminotransferase, ALT: alanine aminotransferase, APRI: AST-to-platelet ratio index, and FIB4: fibrosis-4.

Based on serological findings, HIV patients were classified into four groups: HIV, HIV/HBsAg, HIV/HCV, and HIV/HCV/HBsAg, as shown in Figure 3. No significant differences were observed across these groups in the median HIV viral load (*p* = 0.350) and CD8+ counts (*p* = 0.082) (Figure 3A,B). Patients with triple infections (HIV/HCV/HBsAg) exhibited the lowest CD4+ counts (223.0 cells/mm^3^; IQR: 89–352.0 cells/mm^3^; *p* = 0.22) (Figure 3C). Furthermore, the HIV/HCV group had the highest levels of AST and ALT, with medians of 38.0 IU/mL (IQR: 31.0–60.0 IU/mL) and 36.0 IU/mL (IQR: 24–60.0 IU/mL), respectively (Figure 3D,E). Both the median of APRI and liver stiffness exhibited a significant elevation in patients with HIV/HCV in comparison with those in the HIV or HIV/HBV groups (APRI: 0.5, IQR = 0.3–0.8 vs. 0.3, IQR: 0.2–0.6, *p* = 0.0053, or APRI: 0.3, IQR: 0.2–0.5, *p* = 0.0018; liver stiffness: 1.2 kPa, IQR: 0.9–2.3 kPa vs. 1.1 kPa, IQR: 0.7–1.8 kPa, *p* = 0.016, or liver stiffness: 0.9 kPa, IQR: 0.7–1.2 kPa, *p* = 0.0066) (Figure 3F,H). Similarly, the median FIB4 was notably higher in the HIV/HCV group compared with the HIV/HBsAg group (1.2; IQR: 0.9–2.3 vs. 0.9; IQR: 0.7–1.8; *p* = 0.0018) (Figure 3G).

The overall prevalence of advanced liver fibrosis (F3–F4) was 32.9% (46/140). Notably, the highest prevalence of advanced liver fibrosis (F3–F4) was found in the HIV/HCV group at 49.2% (30/61), followed by HIV/HCV/HBsAg at 25.0% (1/4), HIV at 23.8% (5/21), and HIV/HBsAg at 18.5% (10/54). This trend remained consistent when the APRI and FIB4 were analyzed. The HIV/HCV group showed a significantly higher prevalence of advanced liver fibrosis (F3–F4) compared with the HIV/HBsAg group (49.2%, 30/61 vs. 18.5%, 10/54; *p* = 0.00465). The prevalence of advanced liver fibrosis (F3–F4) in patients with evidence of HCV co-infection (HIV/HCV + HIV/HCV/HBsAg) was of 47.7% (31/65).

### 3.5. Liver Fibrosis by HCV Subtype

**Figure 4:**
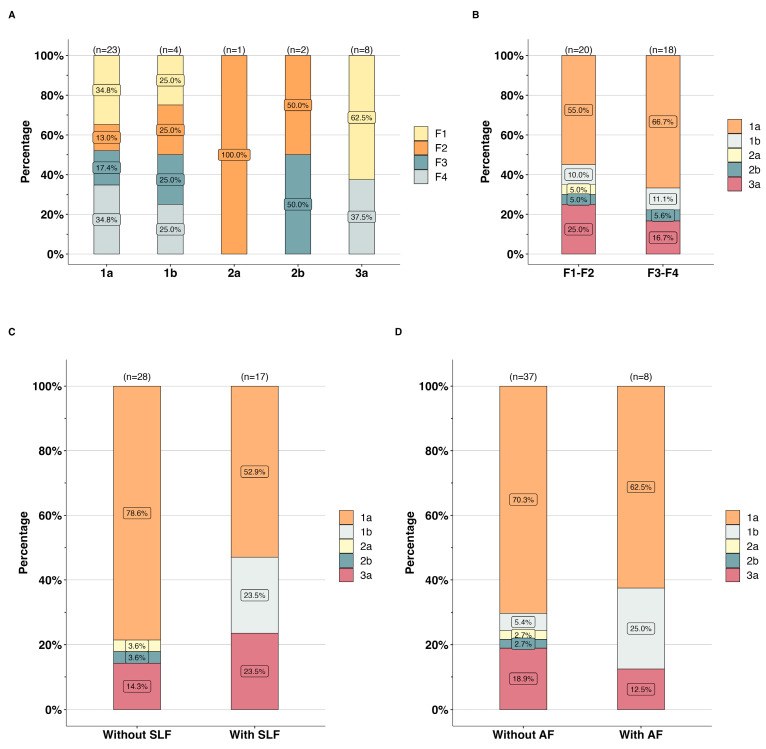
**(A)** Liver fibrosis degree by HCV subtypes in patients with HIV. Distribution of HCV subtypes among patients with (**B**) advanced liver fibrosis (F3–F4), (**C**) significant liver fibrosis (SLF), and (**D**) advanced fibrosis (AF).

The frequency of advanced liver fibrosis (F3–F4) tended to differ between hepatitis C subtypes, as shown in Figure 4. The most prevalent hepatitis C subtype in patients with advanced liver fibrosis (F3–F4) was 1a (66.7%, 12/18), followed by 3a (16.7%, 3/18), 1b (11.1%, 2/18), and 2b (5.6%, 1/18) (Figure 4A,B). Using the APRI and FIB4, HCV subtypes 1a, 1b, and 3a were predominant in patients with significant liver fibrosis (52.9%, 9/17; 23.5%, 4/17; and 23.5%, 4/17) or with advanced liver fibrosis (62.8%, 5/8; 25.0%, 2/8; and 12.5%, 1/8) (Figure 4C,D), respectively.

### 3.6. Risk Factors Associated with Liver Fibrosis in HIV Patients

We also studied the risk factors associated with advanced liver fibrosis (F3–F4), significant liver fibrosis (APRI), and advanced fibrosis (FIB4) in patients with HIV (Table 2, Table 3 and Table 4). Based on liver stiffness, HIV/HCV co-infection increased the risk of advanced liver fibrosis (F3–F4) 4.3-fold (95% CI: 1.9–10.4; *p* = 0.001) compared with HIV/HBsAg co-infected patients. Also, the univariate analysis identified age ≥ 41.5 years (OR = 2.7; 95% CI: 1.3–5.7; *p* = 0.007), direct bilirubin ≥ 0.16 mg/dL (OR = 3.8; 95% CI: 1.6–9.3; *p* = 0.003), and AST ≥ 98.0 IU/L (OR = 7.8; 95% CI: 2.1–37.7; *p* = 0.004) as risk factors associated with advanced liver fibrosis (F3–F4) in the HIV cohort (Table 2). Using the parameters of the multivariate binary logistic regression (β0 = −1.591, β1 = 1.945, and β2 = 1.077), we calculated that a patient co-infected with HIV/HCV and AST ≥ 98.0 IU/L had an 80.7% likelihood of presenting advanced liver fibrosis (F3–F4).

Biochemical parameters such as direct bilirubin ≥ 0.16 mg/dL (OR = 7.8; 95% CI: 3.8–16.8; *p* < 0.001), indirect bilirubin ≥ 0.78 mg/dL (OR = 4.0; 95% CI: 1.9–8.5; *p* < 0.001), ALT ≥ 54.5 IU/L (OR = 13.8; 95% CI: 6.5–30.7; *p* < 0.001), GGT ≥ 70.5 IU/L (OR = 3.7; 95% CI: 1.8–7.7; *p* < 0.001), albumin ≤ 3.4 g/dL (OR = 6.7; 95% CI: 3.3–14.3; *p* < 0.001), CD4+ counts ≤ 191.5 cells/mm^3^ (OR = 5.3; 95% CI: 2.6–10.8; *p* < 0.001), and the presence of HCV infection (OR = 2.4; 95% CI: 1.0–6.1; *p* = 0.050) were identified as individual risk factors associated with significant liver fibrosis (Table 3).

The APRI variables associated with liver fibrosis were similar to those identified by FIB4 analysis (Table 4). Notably, the presence of hepatitis C (OR = 4.0; CI: 1.2–18.3; *p* = 0.041), direct bilirubin ≥ 0.20 mg/dL (OR = 24.0; 95% CI: 7.5–106.8; *p* < 0.001), indirect bilirubin ≥ 0.78 mg/dL (OR = 8.3; 95% CI: 3.2–23.0; *p* < 0.001), and GGT ≥ 82.5 IU/L (OR = 5.7; 95% CI: 2.2–15.7; *p* < 0.001), as well as low levels of albumin of ≤3.7 g/dL (OR = 21.2; 95% CI: 4.2–386.7; *p* = 0.003) and CD4 counts ≤ 191.5 cells/mm^3^ (OR = 5.8; 95% CI: 2.2–16.3; *p* < 0.001), were identified as significant risk factors for advanced fibrosis in HIV patients.

## 4. Discussion

A high prevalence of HCV infection (36.4%) was found among HIV patients in West Mexico, surpassing national rates of HIV/HCV co-infection (2.5%) and those reported in other regions of Mexico, such as the central (6.1%) and northern (12.1%) areas [11,24,25,26]. Moreover, the prevalence in West Mexico exceeded that reported in other countries, including the US (25.0%), Malaysia (16.1%), and Brazil (6.9%) [27,28,29]. In this study, we identified regional factors contributing to the high HIV/HCV co-infection rate. One of the most significant factors was IDU, a practice directly influenced by the extensive drug trafficking in the western region [7]. This factor is also closely related to the number of people imprisoned in Mexico. In 2012, it was estimated that 60% of individuals confined in federal prisons were arrested for drug-related crimes, such as trafficking, consumption, and illegal possession [30]. In 2022, illicit drug possession was the seventh most common criminal behavior among youth (aged 16 to 22) in Mexican prisons [31]. These findings may, in part, explain why HIV-infected people with a history of incarceration had a 3.02-fold increased risk of HIV/HCV co-infection compared with those without such a history. These findings suggest a complex interaction between drug trafficking, imprisonment, and viral hepatitis, underscoring the urgent need to develop effective strategies to address hepatitis C in Mexico and advance toward the World Health Organization’s goals of eliminating hepatitis C infection by the year 2030 [32,33].

Socioeconomic factors may also impact the risk for HIV/HCV co-infection. In West Mexico, 84.0% of HIV patients live in poverty [23], and 77% of the cohort of HIV/HCV co-infected patients had primary or no education. Poverty and limited education can lead to high-risk behaviors for viral hepatitis, such as those associated with sex and drugs [9]. Herein, sex workers had a 1.65 times higher risk of HIV/HCV co-infection compared with other occupations. Lack of education may also contribute to risky behaviors, such as early onset of sexual activity and unprotected sexual activities [34,35]. This study revealed an association between the onset of sexual activity before the age of 17.5 years and the prevalence of HIV/HCV co-infection. The high prevalence of HCV/HIV co-infection may be linked to the prevalence of unconventional sexual activities, such as “receptive fisting” and “chemsex” [36].

Besides drug use, this study linked HIV/HCV co-infection to blood transfusions and surgeries. Historically, medical interventions caused blood-borne infections [37,38,39,40]. In 1993, Mexican officials installed safe blood donation programs [41,42]; however, the risk of transmission of viral infections through blood transfusions or surgical supplies may persist in healthcare environments lacking essential infrastructure and proper sterilization equipment [43]. This problem could also affect tattoo establishments since tattooed individuals had 2.35 times the risk of HIV/HCV co-infection. A meta-analysis that examined 83 studies worldwide confirmed the strong association between tattoos and HCV infection [44]. Implementing hygiene/sterility certifications and laboratory requirements before tattooing could mitigate the risk of viral hepatitis transmission in tattoo establishments. Additionally, an adaptable healthcare system that strengthens infrastructure and provides training for health professionals is crucial for the long-term control and prevention of viral hepatitis in developing countries like Mexico.

Currently, HCV is classified into eight genotypes; each is further divided into subtypes identified by a combination of numbers and letters [45,46]. Genotype 1, which includes subtypes 1a and 1b, is the most prevalent, followed by genotypes 3, 4, and 2 [47]. Genotypes 5 and 6 are less common [47]. In 2015, Canada isolated the first sequence of HCV genotype 7 from an emigrant from the Democratic Republic of Congo [48]. In 2018, four patients with the new genotype 8 were identified in the Punjab region, India [49]. Recently, HCV subtypes/genotypes 1a, 1b, 2, 3, 4, and 5 have been reported among risk groups in Mexico [24]. In this study, the distribution of HCV subtypes among HIV/HCV co-infected individuals in West Mexico was 1a, 3a, 1b, 2b, 2a, and 4a. Notably, HCV subtype 1a was the most prevalent, at 68.2%. This finding aligns with the rate observed in the central region of the country (65.6%) [36]. This similarity suggests a potential link with the mobility of HIV patients. Phylodynamic research has shown continuous migration between central Mexico and Jalisco (West Mexico), Quintana Roo (Southern Mexico), and Baja California Norte (Northern Mexico) [50]. The authors also reported that HIV patients primarily travel to California in the US (75%) [50]. Probably, the migration pattern of HIV patients, as well as immigrants from other regions of the world, could introduce HCV less-common genotypes to Mexico, such as HCV genotype 4 or 5.

Alcoholic liver disease (45%) and HCV infection (43%) are the leading causes of end-stage liver disease in Mexico [51]. Among HIV patients, 32.9% showed advanced liver damage (F3–F4), with the highest prevalence seen in patients with HIV/HCV co-infection (47.7%). Globally, advanced liver damage rates in patients with HIV/HCV co-infection range from 25% to 40% [52,53], suggesting our prevalence is among the highest worldwide. In addition to the socioeconomic and geographic factors mentioned above, the high prevalence of advanced liver damage in patients with HIV/HCV co-infection could be attributed to the combination of other factors. Notably, the lack of sensitive tools to assess the degree of liver fibrosis in HIV units could result in delayed diagnoses of liver damage. In risk groups without HIV, the average age of patients with advanced liver damage was 48.9 years [51], whereas in this study, the age significantly associated with advanced liver damage was 41.5 years. Therefore, liver fibrosis might manifest approximately 7.1 years earlier in HIV/HCV co-infected individuals compared with other patients with chronic liver diseases in West Mexico. Furthermore, hepatic damage could occur even earlier in some regions [54], underscoring the need for routine liver fibrosis assessments in HIV clinics. The effectiveness of this measure has been demonstrated in the US, where treating HCV in the early stages of fibrosis improved health outcomes and reduced long-term management costs [55].

The distribution of HCV subtypes among the HIV cohort may significantly contribute to liver damage prevalence, especially subtypes 1a, 1b, and 3a. In this study, subtype 1a was most prevalent among HIV patients with advanced liver damage (66.7%), significant liver fibrosis (52.9%), or advanced fibrosis (62.5%). This subtype is known to be susceptible to resistance-associated substitutions (RASs) in the non-structural protein 5A (NS5A) [56], while subtype 1b has been associated with hepatocellular carcinoma in patients with cirrhosis [57]. In this study, HCV subtype 3a was the second most common (16.7%) among HIV patients with advanced liver damage. In 2009, this subtype was associated with an accelerated progression of fibrosis [58]. Two years later, this association was confirmed through a meta-analysis that examined 16 studies based on liver biopsies [59]. Moreover, HCV subtype 3 has been linked to high viral load levels and is associated with severe clinical outcomes, such as end-stage liver disease, hepatocellular carcinoma, and liver-related mortality [60,61]. Among the major HCV subtypes, subtype 3a can present low success rates of direct-acting antivirals at 12 weeks [62]. Recently, a valine mutation at position 132 in the NS2 protein significantly reduced the response to treatment [63], which could partly explain why some patients with HCV subtype 3 require more extended therapy periods [64]. Furthermore, a sustained virological response can be influenced by a combination of factors, including the HCV subtype and patient characteristics such as age, polymorphisms, comorbidities, and the stage of liver disease [65,66,67,68].

We explored additional parameters for liver fibrosis, finding CD4+ levels of ≤191.5 cells/mm^3^ linked to significant or advanced fibrosis. Previous studies associated CD4+ levels of <350 cells/µL with hepatic events in patients with HIV/HCV co-infection and of <200 cells/mm^3^ with liver fibrosis in HIV patients [69,70,71,72]. However, exact CD4+ levels for liver fibrosis have not been standardized. Another parameter associated with liver fibrosis was low albumin levels [73,74]. Previously, this condition has been associated with cirrhosis in HCV patients [75]. Albumin levels of <4 g/dL can increase the risk of death 13.3-fold in patients with compensated cirrhosis [76], and levels of <3.5 g/dL are linked to rapid HIV progression [77]. In the HIV cohort, albumin levels of ≤3.4 g/dL and ≤3.7 g/dL indicated significant and advanced fibrosis. An often-present symptom in patients with liver damage is jaundice, characterized by yellowing of the skin or sclera [78]. Typically, jaundice is observed when bilirubin levels exceed 2.5 mg/dL [79]. Studies have reported an association between bilirubin levels and the severity of liver fibrosis in viral hepatitis infections [80,81,82]. Similarly, in this study, elevated levels of indirect bilirubin (≥0.78 mg/dL) and direct bilirubin (≥0.16 mg/dL) were often associated with significant liver fibrosis or advanced fibrosis in HIV patients. Although hyperbilirubinemia alone is not an exclusive symptom of liver diseases [83,84,85], its interpretation, together with other parameters, such as CD4+ levels, albumin levels, platelets, and elevated liver enzymes, could help healthcare professionals identify HIV patients at risk for liver complications. Future studies must evaluate whether clinicians worldwide can use the cut-off values for CD4+, albumin, direct bilirubin, and indirect bilirubin estimated in this study in other HIV cohorts.

Despite its comprehensive approach, this study had some limitations to consider when interpreting the results. HIV-positive participants were recruited from a single hospital situated in West Mexico, which predominantly serves individuals with a low income. Like all cross-sectional studies, our research did not establish the cause-and-effect relationship between the factors and clinical outcomes. Furthermore, hepatitis C infection diagnosis was based on serological tests, which may not accurately reflect the current infection status, as individuals with HCV clearance can still maintain a positive serological test. Our study had a relatively low sample size, and data such as alcohol consumption, the prevalence of hepatic steatosis, and a complete registry of medications that may impact the prevalence of liver fibrosis were not assessed.

Nevertheless, this study had several strengths. It was able to describe a part of the complex molecular epidemiology of HCV in HIV patients, offering valuable insights into the prevalence, risk factors, and liver damage. Furthermore, a significant number of the patient’s strains could be genotyped, allowing a deeper understanding of the viral diversity in patients with HIV. In addition, it was possible to identify biochemical parameters with potential utility in clinical management, thereby contributing to the advancement of patient care strategies.

## 5. Conclusions

This study provides comprehensive insights into the molecular epidemiology and clinical characteristics of HCV infection among HIV patients. We found a high prevalence of HIV/HCV co-infection, especially among individuals with a history of IDU, imprisonment, early onset of sexual activity, blood transfusions, tattooing, sex work, and surgery. These risk factors highlight that the current national program to eliminate hepatitis C in Mexico must reinforce detection efforts and establish targeted preventive measures to reach the global World Health Organization’s elimination goal by 2030. Additionally, these risk factors suggest that social programs, as well as initiatives to combat drug trafficking, may also play a crucial role in the global fight against viral hepatitis. Furthermore, the identification of biochemical parameters associated with liver damage highlights the importance of regular monitoring and early intervention to mitigate the progression of liver disease in the HIV-infected population.

## Figures and Tables

**Table 1:** Sociodemographic and clinical characteristics of the study population.

Variable	Category or Measure	Total (N = 294)	HIV (N = 187)	HIV/HCV (N = 107)	*p*-Value
Gender	Female	35 (11.9%)	20 (10.7%)	15 (14.0%)	0.455 ^1^
	Male	259 (88.1%)	167 (89.3%)	92 (86.0%)	0.397 ^1^
Age (year)	Median (Q1, Q3)	38.0 (32.2, 46.8)	38.0 (31.0, 46.2)	39.5 (34.2, 46.8)	0.131 ^2^
Onset of sexual life	Median (Q1, Q3)	17.0 (15.0, 18.0)	18.0 (15.0, 19.0)	15.0 (13.0, 18.0)	<0.001 ^2^
Sexual preference	Heterosexual	121 (43.1%)	64 (36.2%)	57 (54.8%)	<0.001 ^1^
	Bisexual	61 (21.7%)	41 (23.2%)	20 (19.2%)	0.459 ^1^
	Homosexual	99 (35.2%)	72 (40.7%)	27 (26.0%)	0.008 ^1^
Education	High school/university	101 (35.6%)	77 (43.0%)	24 (22.9%)	<0.001 ^1^
	Basic/none	183 (64.4%)	102 (57.0%)	81 (77.1%)	<0.001 ^1^
Hepatitis B status	HBsAg (−)/anti-HBc (−)	149 (50.7%)	84 (44.9%)	65 (60.7%)	0.012 ^1^
	HBsAg (−)/anti-HBc (+)	55 (18.7%)	21 (11.2%)	34 (31.8%)	<0.001 ^1^
	HBsAg (+)/anti-HBc (−)	13 (4.4%)	12 (6.4%)	1 (0.9%)	0.036 ^1^
	HBsAg (+)/anti-HBc (+)	53 (18.0%)	49 (26.2%)	4 (3.7%)	<0.001 ^1^
	HBsAg (+)/anti-HBc not tested	24 (8.2%)	21 (11.2%)	3 (2.8%)	<0.001 ^1^
CD8+ cells/mm^3^	Median (Q1, Q3)	895.0 (585.0, 1187.0)	884.0 (599.5, 1212.0)	917.0 (560.0, 1126.5)	0.555 ^2^
CD4+ cells/mm^3^	Median (Q1, Q3)	315.0 (172.0, 531.0)	316.0 (198.2, 543.0)	314.0 (135.5, 509.5)	0.707 ^2^
ALT IU/L	Median (Q1, Q3)	28.0 (21.0, 50.0)	27.0 (20.0, 40.0)	36.0 (24.0, 59.0)	0.004 ^2^
AST IU/L	Median (Q1, Q3)	32.0 (25.0, 48.0)	29.0 (24.0, 40.0)	38.0 (30.5, 60.5)	<0.001 ^2^
GGT IU/L	Median (Q1, Q3)	44.0 (26.2, 77.8)	39.0 (25.0, 60.0)	55.0 (31.0, 175.0)	0.003 ^2^
Platelet cells/µL	Median (Q1, Q3)	219.0 (174.8, 279.0)	229.0 (184.0, 289.0)	208.0 (151.5, 263.0)	0.094 ^2^
HIV viral load (copies/mL)	Median (Q1, Q3)	40.0 (34.8, 225.2)	40.0 (31.0, 213.0)	42.0 (40.0, 225.5)	0.422 ^2^
HIV viral load class *	Unavailable	78 (26.5%)	50 (26.7%)	28 (26.2%)	1.000 ^1^
	Suppressed < 1000 copies/mL	169 (57.5%)	104 (55.6%)	65 (60.7%)	0.463 ^1^
	Unsuppressed ≥ 1000 copies/mL	47 (16.0%)	33 (17.6%)	14 (13.1%)	0.388 ^1^
HCV viral load (IU/mL)	Median (Q1, Q3)	2,165,000.0 (106,374.2, 11,800,000.0)	-	2,165,000.0 (106,374.2, 11,800,000.0)	

^1^ Chi-squared or Fisher’s exact test; ^2^ Mann–Whitney test; * stratification of HIV viral load according to the World Health Organization’s classification: https://www.who.int/publications/i/item/9789240055179, accessed on 8 April 2024.

**Table 2:** Risk factors associated with advanced liver fibrosis among patients with HIV.

Liver Stiffness	Variable	F1–F2	F3–F4	OR (Univariable)	OR (Multivariable)
Age (year)	Age < 41.5	63 (67.7)	20 (43.5)	-	-
	Age ≥ 41.5	30 (32.3)	26 (56.5)	2.73 (1.33–5.71; *p* = 0.007)	-
DB (mg/dL)	DB < 0.16	57 (78.1)	16 (48.5)	-	-
	DB ≥ 0.16	16 (21.9)	17 (51.5)	3.79 (1.58–9.28; *p* = 0.003)	-
AST (IU/L)	AST < 98.0	73 (96.1)	25 (75.8)	-	-
	AST ≥ 98.0	3 (3.9)	8 (24.2)	7.79 (2.08–37.70; *p* = 0.004)	9.75 (2.01–72.88; *p* = 0.009)
Viral hepatitis	HIV/HBsAg	44 (58.7)	10 (25.0)	-	-
	HIV/HCV	31 (41.3)	30 (75.0)	4.26 (1.87–10.36; *p* = 0.001)	3.69 (1.38–10.79; *p* = 0.012)

AST: aspartate aminotransferase, DB: direct bilirubin, F3–F4: advanced liver fibrosis, F1–F2: mild liver fibrosis, HIV: human immunodeficiency virus, HBsAg: hepatitis B surface antigen, and HCV: hepatitis C virus. The cut-off values were calculated using ROC curve analysis.

**Table 3:** Risk factors associated with significant liver fibrosis among patients with HIV.

APRI	Variable	Without SLF	With SLF	OR (Univariable)	OR (Multivariable)
Platelet cells/µL	Platelets > 157.5	155 (95.1)	14 (29.8)	-	-
	Platelets ≤ 157.5	8 (4.9)	33 (70.2)	45.67 (18.60–125.18; *p* < 0.001)	-
DB (mg/dL)	DB < 0.16	120 (75.5)	13 (28.3)	-	-
	DB ≥ 0.16	39 (24.5)	33 (71.7)	7.81 (3.82–16.81; *p* < 0.001)	-
IB (mg/dL)	IB < 0.78	137 (86.2)	28 (60.9)	-	-
	IB ≥ 0.78	22 (13.8)	18 (39.1)	4.00 (1.90–8.46; *p* < 0.001)	5.12 (1.65–17.00; *p* = 0.005)
ALT (IU/L)	ALT < 54.5	146 (89.6)	18 (38.3)	-	-
	ALT ≥ 54.5	17 (10.4)	29 (61.7)	13.84 (6.51–30.74; *p* < 0.001)	16.56 (6.30–48.58; *p* < 0.001)
AST (IU/L)	AST < 43.5	141 (86.5)	6 (12.8)	-	-
	AST ≥ 43.5	22 (13.5)	41 (87.2)	43.80 (17.78–126.25; *p* < 0.001)	-
GGT (IU/L)	GGT < 70.5	120 (78.9)	22 (50.0)	-	-
	GGT ≥ 70.5	32 (21.1)	22 (50.0)	3.75 (1.85–7.67; *p* < 0.001)	-
Albumin (g/dL)	Albumin > 3.4	124 (80.0)	16 (37.2)	-	-
	Albumin ≤ 3.4	31 (20.0)	27 (62.8)	6.75 (3.29–14.32; *p* < 0.001)	5.60 (2.04–16.69; *p* = 0.001)
CD4+ cells/mm^3^	CD4+ > 191.5	130 (80.2)	20 (43.5)	-	-
	CD4+ ≤ 191.5	32 (19.8)	26 (56.5)	5.28 (2.64–10.76; *p* < 0.001)	3.41 (1.27–9.48; *p* = 0.016)
Viral hepatitis	HIV/HBsAg	56 (53.3)	9 (32.1)	-	-
	HIV/HCV	49 (46.7)	19 (67.9)	2.41 (1.02–6.06; *p* = 0.050)	-

Variables used to calculate APRI were excluded from the multivariate analysis, and non-significant variables were excluded from the univariate analysis. ALT: alanine aminotransferase, AST: aspartate aminotransferase, GGT: gamma-glutamyl transferase, APRI: AST-to-platelet ratio index, CD4+: lymphocyte CD4+ count, DB: direct bilirubin, IB: indirect bilirubin, SLF: significant liver fibrosis, HIV: human immunodeficiency virus, HBsAg: hepatitis B surface antigen, and HCV: hepatitis C virus. The cut-off values were calculated using ROC curve analysis.

**Table 4:** Risk factors associated with advanced fibrosis among patients with HIV.

FIB4	Variable	Without AF	With AF	OR (Univariable)	OR (Multivariable)
Platelet cells/µL	Platelets > 124.5	179 (95.7)	3 (14.3)	-	-
	Platelets ≤ 124.5	8 (4.3)	18 (85.7)	134.25 (37.04–668.41; *p* < 0.001)	-
DB (mg/dL)	DB < 0.20	148 (80.9)	3 (15.0)	-	-
	DB ≥ 0.20	35 (19.1)	17 (85.0)	23.96 (7.55–106.76; *p* < 0.001)	-
IB (mg/dL)	IB < 0.78	155 (84.7)	8 (40.0)	-	-
	IB ≥ 0.78	28 (15.3)	12 (60.0)	8.30 (3.16–22.99; *p* < 0.001)	13.32 (4.00–50.49; *p* < 0.001)
ALT (IU/L)	ALT < 47.5	145 (77.5)	7 (33.3)	-	-
	ALT ≥ 47.5	42 (22.5)	14 (66.7)	6.90 (2.69–19.27; *p* < 0.001)	-
AST (IU/L)	AST < 48.5	154 (82.4)	3 (14.3)	-	-
	AST ≥ 48.5	33 (17.6)	18 (85.7)	28.00 (8.86–124.55; *p* < 0.001)	-
GGT (IU/L)	GGT < 82.5	138 (79.3)	8 (40.0)	-	-
	GGT ≥ 82.5	36 (20.7)	12 (60.0)	5.75 (2.22–15.70; *p* < 0.001)	-
Albumin (g/dL)	Albumin > 3.7	102 (57.0)	1 (5.9)	-	-
	Albumin ≤ 3.7	77 (43.0)	16 (94.1)	21.19 (4.19–386.68; *p* = 0.003)	18.98 (3.19–367.86; *p* = 0.007)
CD4+ cells/mm^3^	CD4+ > 191.5	141 (75.8)	7 (35.0)	-	-
	CD4+ ≤ 191.5	45 (24.2)	13 (65.0)	5.82 (2.24–16.33; *p* < 0.001)	4.04 (1.17–15.80; *p* = 0.033)
Viral hepatitis	HIV/HBsAg	61 (52.1)	3 (21.4)	-	-
	HIV/HCV	56 (47.9)	11 (78.6)	3.99 (1.18–18.33; *p* = 0.041)	-

Variables used to calculate FIB4 were excluded from the multivariate analysis, and non-significant variables from the univariate analysis. ALT: alanine aminotransferase, AST: aspartate aminotransferase, GGT: gamma-glutamyl transferase, FIB4: fibrosis-4 score, CD4+: lymphocyte CD4+ count, DB: direct bilirubin, IB: indirect bilirubin, AF: advanced fibrosis, OR: odds ratio, HIV: human immunodeficiency virus, HBsAg: hepatitis B surface antigen, and HCV: hepatitis C virus. The cut-off values were calculated using ROC curve analysis.

## Data Availability

Data are contained within the article.
